# Supplementary material for: High temperature superconductivity at FeSe/LaFeO3 interface
Source: Nat Commun. 2021 Oct 11;12:5926. doi: 10.1038/s41467-021-26201-2 (PMC8505662; doi:10.1038/s41467-021-26201-2)
Supplement: Supplementary file 1 — Supplementary Information [file 41467_2021_26201_MOESM1_ESM.pdf]

Supplementary Information for "High temperature superconductivity at FeSe/$LaFeO_3$ interface"

Yuanhe Song,[1] Zheng Chen,[2] Qinghua Zhang,[3] Haichao Xu,[1,4] Xia Lou,[1] Xiaoyang Chen,[1] Xiaofeng Xu,[3] Xuetao Zhu,[3] Ran Tao,[1] Tianlun Yu,[1] Hao Ru,[1] Yihua Wang,[1,4] Tong Zhang,[1,4] Jiandong Guo,[3,*] Lin Gu,[3,†] Yanwu Xie,[2,‡] Rui Peng,[1,4,§] and Donglai Feng[5,4,6,¶]

[1]Laboratory of Advanced Materials, State Key Laboratory of Surface Physics, and Department of Physics, Fudan University, Shanghai 200438, China
[2]Department of Physics, Zhejiang University, Hangzhou 310027, China
[3]Beijing National Laboratory for Condensed Matter Physics and Institute of Physics, Chinese Academy of Sciences, Beijing 100190, China
[4]Shanghai Research Center for Quantum Sciences, Shanghai 201315, China
[5]Hefei National Laboratory for Physical Science at Microscale and Department of Physics, University of Science and Technology of China, Hefei, Anhui 230026, China
[6]Collaborative Innovation Center of Advanced Microstructures, Nanjing 210093, China

(Dated: September 15, 2021)

Contents

Supplementary Note 1. Details for sample preparation and experimental methods 2

Supplementary Note 2. Additional data from cross-sectional STEM 3

Supplementary Note 3. Avoiding photoemission charging effect and superconducting gap determination of 1uc FeSe/LFO/STO 4

Supplementary Note 4. ARPES data of another 1uc FeSe/6uc LFO/STO sample without charging effect 5

Supplementary Note 5. Comparison of sample quality 6

Supplementary Note 6. Band structure of 1.5uc FeSe/LFO/STO 7

Supplementary Note 7. Simulation of partial gap opening 8

Supplementary Note 8. Analysis of the intensity ratio of the replica bands to main bands 9

References 9

SUPPLEMENTARY NOTE 1. DETAILS FOR SAMPLE PREPARATION AND EXPERIMENTAL METHODS

In order to get STO with atomically flat TiO_x terminated surface, Nb-doped STO (0.5%wt) substrates were first etched with buffered-oxide etchant to remove the strontium hydroxide and then annealed in air at 950 °C for 150 min. 6uc of $LaFeO_3$ films were deposited on STO by the pulsed laser deposition (PLD). STO substrates were pre-annealed at 975 °C, and then LFO were grown at 800 °C under an oxygen pressure of 1×10^{-4} mbar. During the deposition, the substrate surface was monitored by a reflective high-energy electron diffractometer (RHEED) and the layer-dependent oscillations of the intensity of the diffracted spots were observed [Supplementary Fig. 1(a)-(b)]. After that, the as-grown LFO/STO were annealed at 600 °C under 200 mbar oxygen pressure for 1 h. Supplementary Fig. 1(c) shows the x-ray reflectivity measurements(XRR) on the LFO/STO samples in the main text. The thickness of the LFO grown on STO was determined to be 2.4 nm, consistent with the nominal thickness of 6uc.

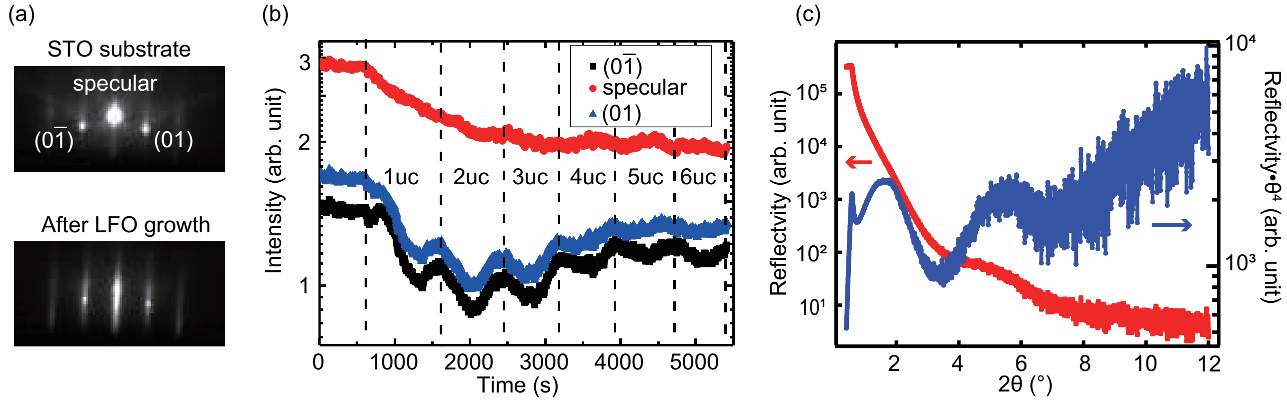

Supplementary Figure 1. Characterization of LFO/STO. (a) RHEED pattern before and after the growth of $LaFeO_3$ on $SrTiO_3$ by PLD method. (b) The time-dependent intensity evolution of the specular, $(0\bar{1})$ and (01) spots/streaks. (c) The spectrum of x-ray reflectivity (red) *v.s.* two-theta. The reflectivity times θ^4 (blue) is for enhancing visibility of the oscillation.

The insulating behavior of $LaFeO_3$ can induce poor grounding and cause severer photoemission charging effect to the ARPES studies on the epitaxial FeSe film. We improved the grounding by sputtering gold film onto the backside and edges of the 6uc LFO/STO substrate and applying silver paste surrounding the substrate. Then the LFO/STO heterostructure was transferred to another vacuum chamber for FeSe growth by the molecular beam epitaxy (MBE) method at a base pressure of 7×10^{-10} mbar. It was degassed at 550 °C for 2 h, and then heated to 950 °C under the Se flux for 45 min. Fe and Se were co-deposited to the substrate at a growth temperature of 490 °C with the Se flux twenty times to the Fe flux. After growth, the film was annealed at 520 °C in vacuum for 3 h and directly transferred into the ARPES chamber for ARPES measurements.

For consistency, all data shown in the main text were collected on the same sample. The ARPES data were measured after 1uc and 1.5uc FeSe deposition and post-annealing. Each ARPES measurement was followed by a *in situ* topography measurement using a RHK STM within a combined ultra-high vacuum system. After all the ARPES and STM measurements, the sample was transferred back to the growth chamber and capped with ~40 nm amorphous Se at room temperature for protection. Then it was taken outside the vacuum and measured by a two-coil mutual inductance (MI) studies. After that, the sample was split into two pieces. One of the two pieces was measured by scanning transmission electron microscopy (STEM), while the other was annealed in vacuum to remove the Se capping layers, and then measured by a high-resolution electron energy loss spectroscopy (HREELS).

SUPPLEMENTARY NOTE 2. ADDITIONAL DATA FROM CROSS-SECTIONAL STEM

The lattice parameters are a=3.905 Å for STO and a=3.93 Å (pseudo cubic lattice) for bulk LFO. With such small lattice mismatch of 0.64%, epitaxial 6uc LFO film is expected to be strained to the STO lattice. Consistently, the large scale STEM image (Supplementary Fig. 2) shows that both FeSe film and 6uc LFO are well matched to the lattice of the STO substrate, which suggests both FeSe and LFO are coherently strained to the STO lattice.

Supplementary Fig. 3 shows the annular bright field (ABF) images taken by STEM along STO(100) and (110) directions. By comparing ABF and HAADF images, the oxygen sites that cannot be disclosed in the HAADF images can be better determined.

supplementary Fig. 4 shown the line profiles data across the interface. By analyzing the peak positions, we can get the interlayer distances.

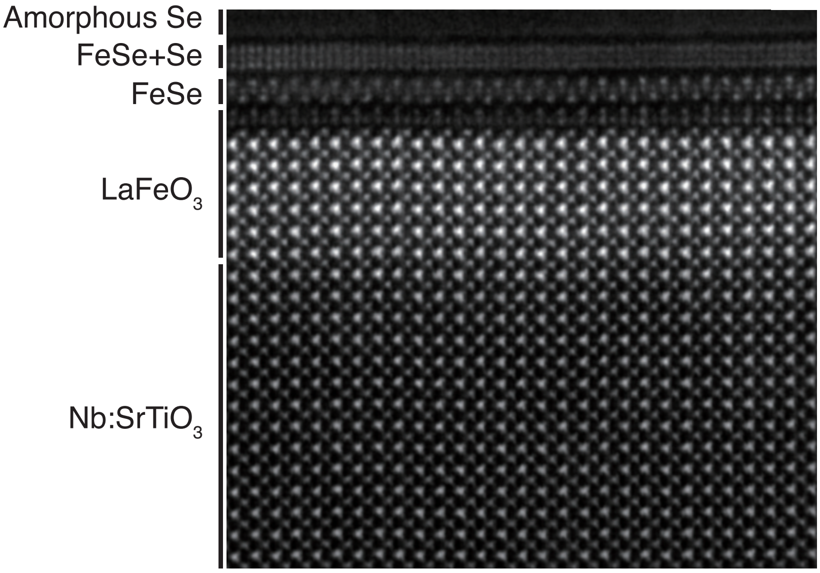

Supplementary Figure 2. STEM-HAADF image of FeSe/LFO/Nb:STO in large scale. STEM-HAADF image of the Se-capped FeSe/LFO/STO sample in a larger scale shows no visible relaxation in the LFO layer.

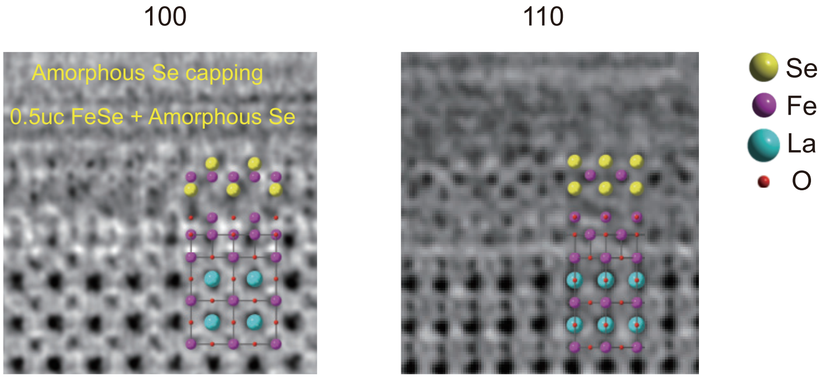

Supplementary Figure 3. STEM-ABF images of the FeSe/LFO interface. Annular bright field (ABF) images taken by STEM along STO(100) and (110) directions.

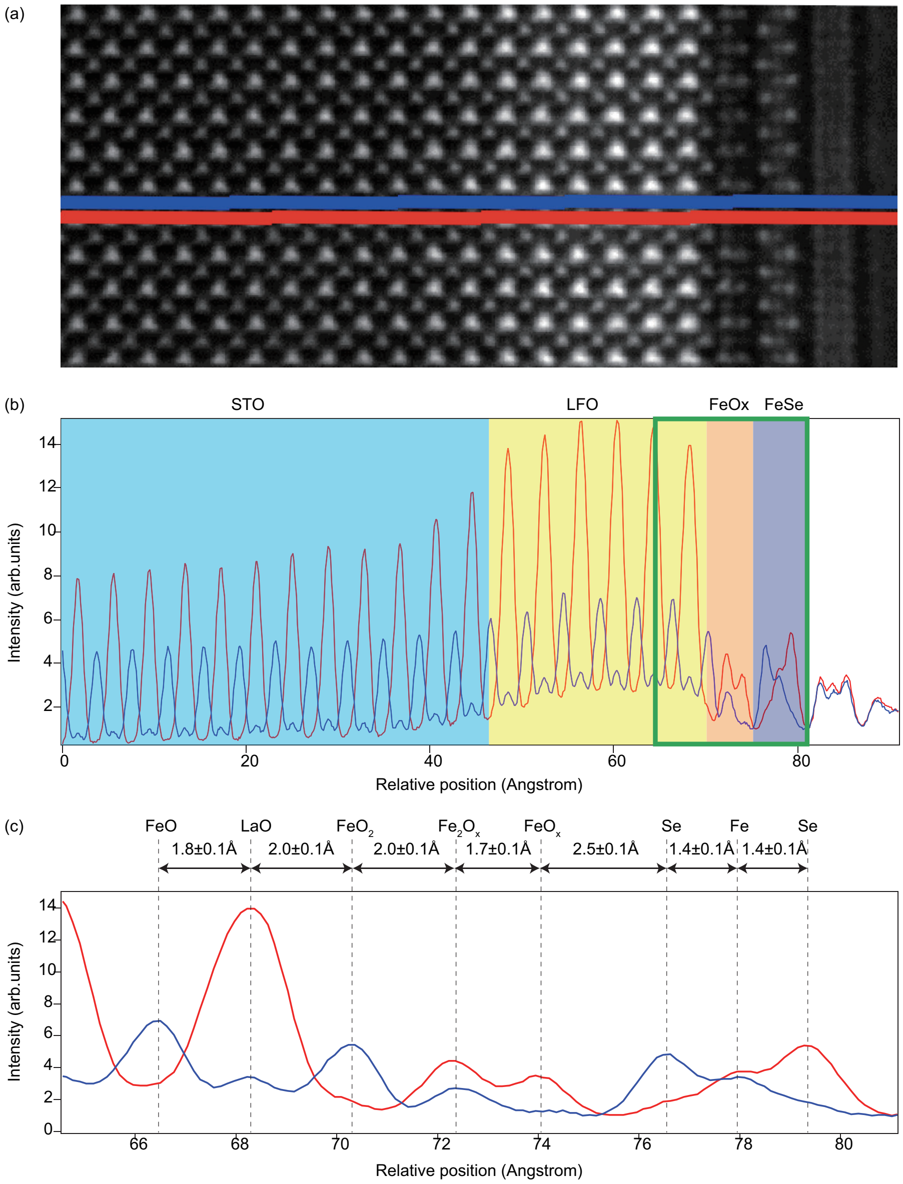

Supplementary Figure 4. Interlayer distance analysis. (a) HAADF image of the selected area with sharp atomic pattern for generating line profiles. (b) Line profiles of the La/Sr atom row (red) and the adjacent Fe/Ti atom row (blue) in panel (a). (c) Zoomed-in view of the line profile inside the green rectangle in panel (b).

SUPPLEMENTARY NOTE 3. AVOIDING PHOTOEMISSION CHARGING EFFECT AND SUPERCONDUCTING GAP DETERMINATION OF 1UC FESE/LFO/STO

It is well known that the charging gap decreases with reduced photon flux density. Therefore, the photon flux has been reduced to eliminate the photoemission charging effect. With I_0, which is the normally used photon flux of our lab-based Helium lamp, a large gap up to 25 meV is observed. The gap decreases with reduced photon flux, indicating an coexistence of charging induced gap in the data measured with I_0 . When the photon flux is reduced to 1/8 I_0, the gap is 17 meV [Figs. 5(b)-(d)], while further decreasing the photon flux to 1/30 I_0, the gap does not change. This indicates a negligible charging effect in the data taken with 1/8 I_0. Besides, by assuming a linear relation between the incident photon flux and the charging induced gap size of the spectra, we estimate that at zero photon flux limit, the gap size is 16.8 meV [Supplementary Fig. 5(d)], representing the intrinsic gap size of this sample.

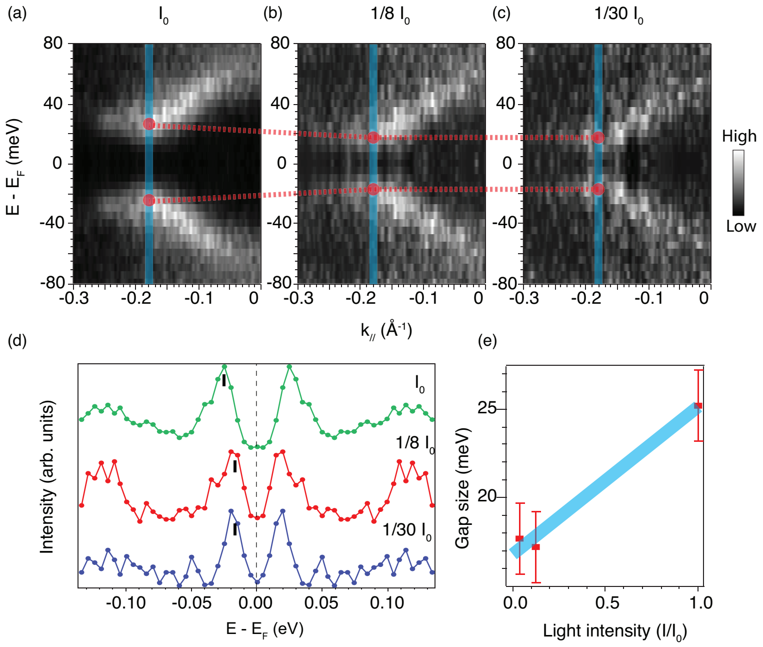

Supplementary Figure 5. Avoiding photoemission charging effect and superconducting gap determination of 1uc FeSe/LFO/STO. (a-c) Symmetrized photoemission spectra of the 1uc FeSe/LFO/STO measured at 6 K with the photon flux of I_0 (a), 1/8 I_0 (b), and 1/30 I_0 (c). The red marks show the coherence peak positions analyzed in panel d. (d) Photon flux dependence of the symmetrized EDCs at k_1, whose momenta is shown by the blue line in panel a-c. (e) Assuming a linear relation between the incident photon flux and the charging induced gap size of the spectra, the superconducting gap in the limit of zero photon flux is 16.8 meV. The error bars of gap are from the s.d. of the fitting process and the measurement uncertainty.

SUPPLEMENTARY NOTE 4. ARPES DATA OF ANOTHER 1UC FESE/6UC LFO/STO SAMPLE WITHOUT CHARGING EFFECT

The measurements of superconducting gap were repeated on different FeSe/LFO/STO samples. supplementary Fig. 6 shows the ARPES data around M point measured on another 1uc FeSe/LFO/STO sample at another ARPES system. At 30 K, the lineshape of the measured EDC is independent of the photon flux used, indicating no photoemission charging effect with the used photon flux I_1. The measured superconducting gap is 17 meV.

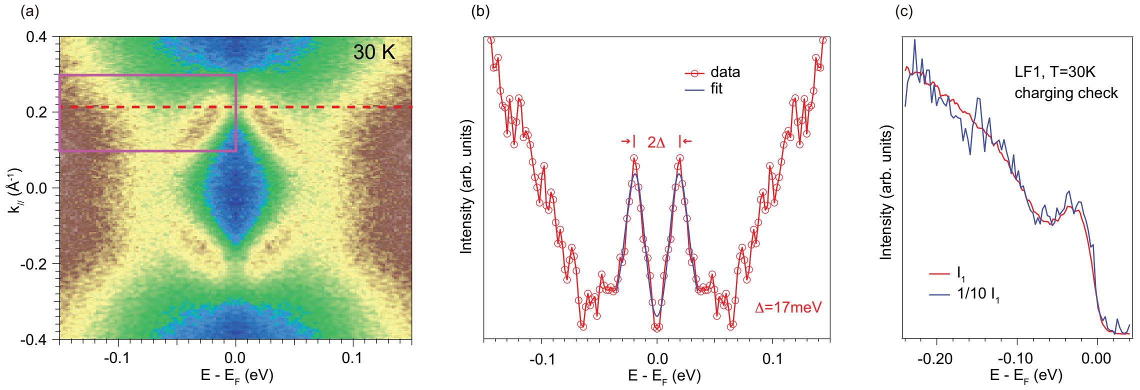

Supplementary Figure 6. ARPES data of another 1uc FeSe/6uc LFO/STO sample. (a) Symmetrized photoemission spectrum of 1uc FeSe/LFO/STO (LF1) measured at 30 K. (b) Symmetrized EDC at the normal state Fermi momentum of band γ_1, whose momenta is shown by the red dashed line in panel a. (c) Integrated EDCs around k_F's measured with different photon flux, showing no photoemission charging effect. The momenta for integration is shown by the pink box in panel a.

SUPPLEMENTARY NOTE 5. COMPARISON OF SAMPLE QUALITY

Impurity scattering which reduces the lifetime of the quasiparticles, can results in the broadening of the ARPES superconducting quasiparticle peak and thus affect the determination of the superconducting gap size. The superconducting gap size can only be compared in samples with similar quality. Here the sample quality of FeSe/LFO/STO is compared with two previous 1uc FeSe/STO samples as shown by the EDCs at normal state Fermi momentum k_1 in Supplementary Fig. 6. The 1uc FeSe/LFO/STO sample shows much sharper coherence peak than an early data on FeSe/STO (ref. 1), and its quality is similar to a more recent data on high quality FeSe/STO sample in ref. 2. It should be noted that the EDCs at k_1 consist of superconducting coherence peaks of the inner electron pocket with gap Δ_1 and spectral weight from the band dispersion of the outer electron pocket. Note that the peak from γ_2 band can be clearly resolved in 1uc FeSe/LFO/STO sample, rather than a dim shoulder in previous reports, which also indicate the superior quality of the 1uc FeSe/LFO/STO sample.

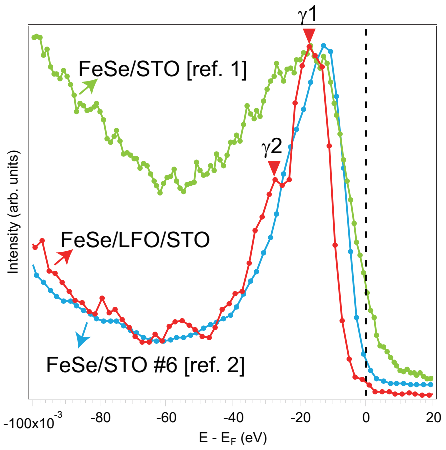

Supplementary Figure 7. Comparison of the superconducting coherence peaks in different samples. EDCs of different samples measured at the normal state Fermi momentum k_1. The momentum of k_1 is shown in Fig. 2e of the main text. The red curve is the data of 1uc FeSe/LFO/STO in the main text. The green curve and the blue curve are from FeSe/STO with 65 K T_g in ref. 1 and #6 in ref. 2. The 1uc FeSe/LFO/STO sample shows sharp superconducting coherence peak similar to the high quality FeSe/STO sample in ref. 2, and much better than the data in the early work (ref. 1). The red triangles mark the superconducting coherence peaks of the inner electron pocket γ_1 and spectral weight from the band dispersion of the outer electron pocket γ_2. The two peaks can be clearly resolved, which also indicate the superior quality of the 1uc FeSe/LFO/STO sample.

The clear identification of the nearly-degenerate bands under the same experimental setup further reflects the high quality of the FeSe/LFO sample. As shown in Supplementary Fig. 8, two nearly degenerate bands can be observed in the 1uc FeSe/LFO presented in our manuscript (noted as Sample #1) and a high-quality 1uc FeSe/STO (noted as Sample #2), but is blurred in a moderate-quality 1uc FeSe/STO (noted as Sample #3), while these data were measured with the same analyzer setup. The peak width reflect the extrinsic broadening from impurity scattering, while the quality of FeSe/LFO is consistent with the FeSe/STO sample with optimized quality to date.

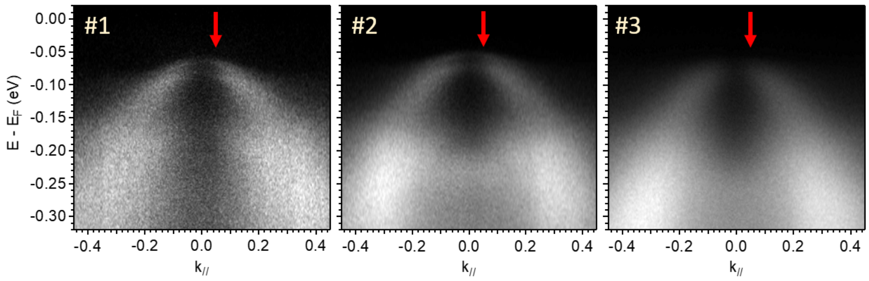

Supplementary Figure 8. Comparison of the band structure near Γ in different samples. The band structure near Γ point of different single layer FeSe samples measured using the same ARPES with same experimental setup. Sample #1, #2 and #3 correspond to the 1uc FeSe/LFO (T=6K), a high-quality 1uc FeSe/STO (T=7K), and a moderate-quality 1uc FeSe/STO (T=8K), respectively. As the red arrows indicated, in both sample #1 and #2, the two nearly-degenerate bands can be clearly observed, while in the sample #3, the two nearly-degenerate bands cannot be distinguished.

SUPPLEMENTARY NOTE 6. BAND STRUCTURE OF 1.5UC FESE/LFO/STO

supplementary Fig. 9 shows the large-energy-scale photoemission spectra of 1.5uc FeSe/LFO/STO. The measured band structure consists of the bands of 1uc FeSe/LFO/STO, together with the weak feature from 2uc FeSe/LFO/STO as shown by the white arrows.

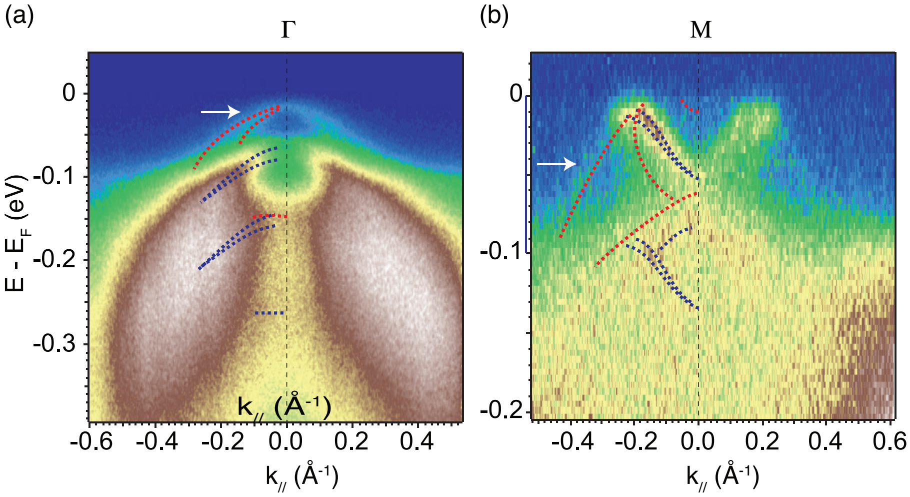

Supplementary Figure 9. Band structure of 1.5uc FeSe/LFO/STO. (a) Photoemission spectrum across Γ of the 1.5 uc FeSe/LFO/STO used in the main text. (b) Same as (a), but across M. The blue dashed curves trace the band dispersions from the 1 uc FeSe portion, and the red dashed curves trace the dispersions from the second-layer FeSe.

SUPPLEMENTARY NOTE 7. SIMULATION OF PARTIAL GAP OPENING

Ideally there should be a partial gap opening between 51 and 80 K as there is a small portion in the sample possessing $T_g \sim 80$ K. However, in experiments with significant thermal broadening and large contributions from normal state regions, the small portion of gap opening would make the symmetrized EDC broader with a flat peak top, and the partial gap opening cannot be observed directly as shown in the following simulation. Supposing that all regions of the sample homogeneously show $T_g \sim 51$ K, there will be no gap at 61 K. As shown in Supplementary Fig. 10(a), we simulate the symmetrized EDC of 61 K with the parameters including Γ_1 and Γ_0 obtained by fitting the 31 K data with a superconducting gap function, and by setting the superconducting gap Δ=0meV and a temperature broadening of 4k$_B$T with T=61K. The measured data is broader than the simulated curve [Supplementary Fig. 10(b)], suggesting an additional broadening in the 61 K data possibly related with a partial gap opening. In Supplementary Fig. 10(c), supposing 1/4 of the sample already opens a gap $\sim$ 17 meV at 61 K, we simulate the superconducting spectral function with Δ=17meV, and adding this spectral function to the Δ=0meV simulated curve with an intensity ratio of 1:3, we can see that the total intensity matches the measured data quite well.

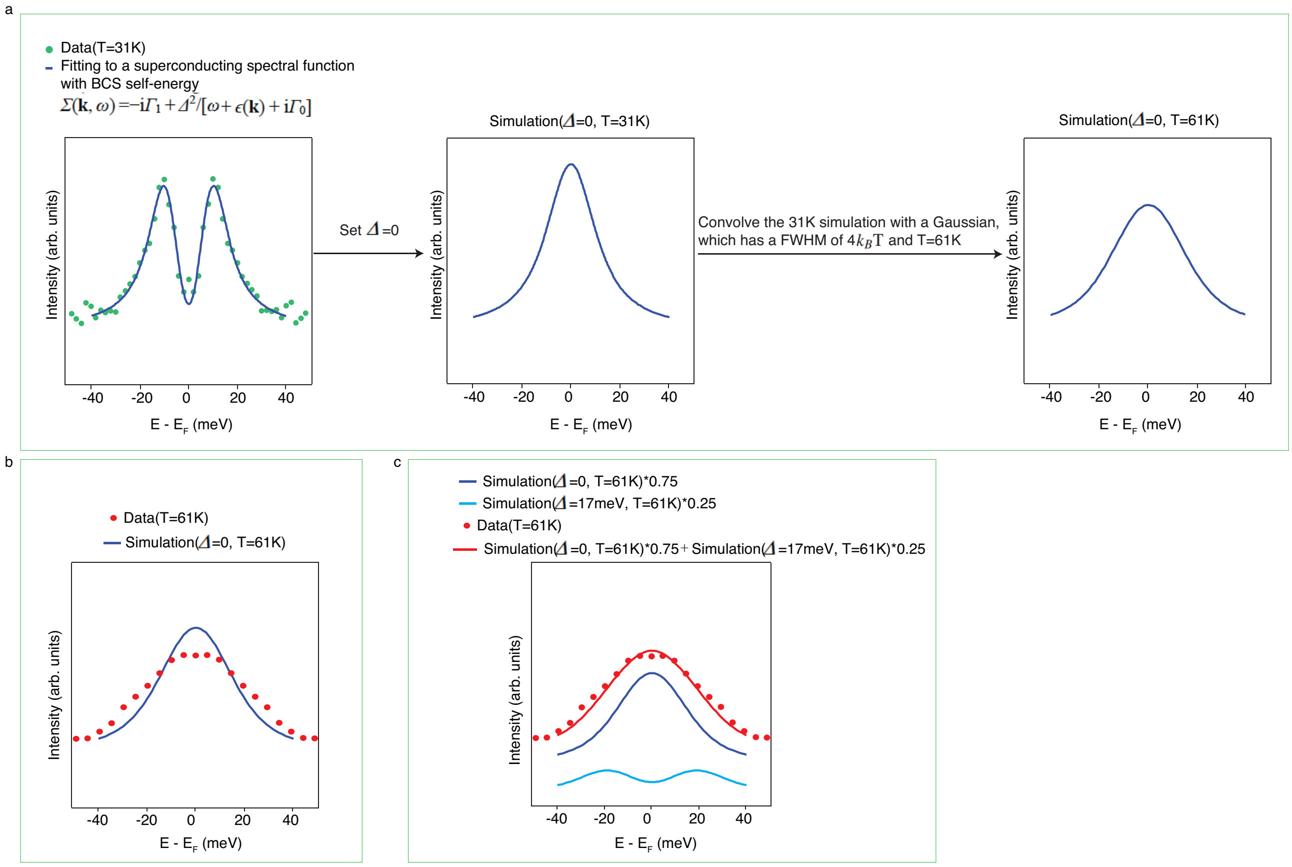

Supplementary Figure 10. Simulation of the partial gap opening at 61 K due to a small portion of sample with $T_g \sim$ 80 K. (a) Left: Symmetrized EDC at 31 K from the Figure 3g in main text and its fitting curve with a superconducting spectral function. Middle: Simulated curve with the fitted parameters including Γ_1 and Γ_0 while the superconducting gap Δ is set to 0 meV. Right: Simulated curve at 61 K obtained by convolving the 31 K simulation by a Gaussian with FWHM of 4k$_B$T (T=61K). (b) Comparison of measured data at 61 K from Figure 3g in main text and the simulation with Δ=0 and T=61K. (c) Comparison of the measured data at 61 K and the simulation considering two contributions of spectral function with from Δ=17meV and Δ=0meV with an intensity ratio of 1:3.

Although the simulation demonstrates that the partial gap opening is probably present, it is not a rigorous demonstration of the ratio of the $T_g \sim 80$ K regions, as the simulation is quite simplified as compared with the real case. For example, the contribution from the superconducting spectral function with Δ=17meV is not considered in the fitting of the 31 K data. Besides, while estimating the portion of superconducting regions, the whole sample should be considered; however, the 1uc FeSe portion that is covered by the second unit cell FeSe in the sample contributes little to ARPES data and its gap size cannot be simulated here.

SUPPLEMENTARY NOTE 8. ANALYSIS OF THE INTENSITY RATIO OF THE REPLICA BANDS TO MAIN BANDS

We analyzed the spectral weight of replica band in Supplementary Fig. 11(a), and the replica band intensity ratio is 0.33 for γ band, and 0.52 for β band. We note that the intensity ratio should be the same for different bands, while here the difference between γ band and β band is probably because the choice of background is not ideal. In FeSe/LFO, the replica band separation is smaller than that in FeSe/STO, and thus the replica band γ' and spectral weight from band β partially overlap (black arrow), making the determination of background more difficult than that in FeSe/STO. In Supplementary Fig. 11, by choosing three different backgrounds, the determined intensity ratio of γ band is different. On the other hand, the intensity ratio of the β band shows a minor influence from the background, this is because that the replica band β' is well separated from other bands, and the intensity ratio of β band should represent the EPC strength more reliably. In FeSe/STO, the replica band β' is hardly observed, while the clear observation of β' in the raw data in Supplementary Fig. 11 indicate a larger EPC in FeSe/LFO. The determined intensity ratio between 0.40 - 0.52, is clearly larger than that in optimized FeSe/STO (~0.2). This is qualitatively consistent with the larger EPC strength determined according to the blue shift of the energy separation relative to the phonon energy.

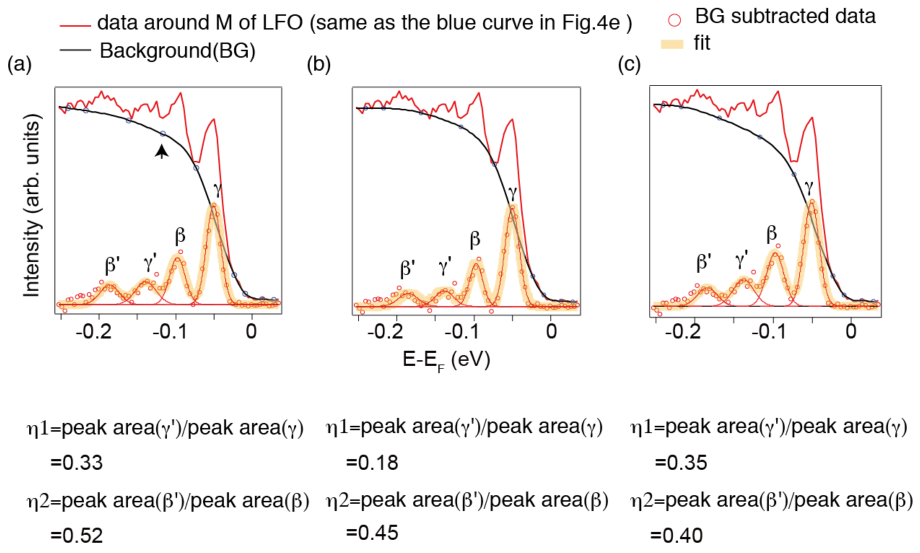

Supplementary Figure 11. Replica band ratio analysis. Analysis of the spectral weight ratio of the replica bands using three different backgrounds. The intensity ratios of the replica band are illustrated below each analysis.

* Electronic address: jdguo@iphy.ac.cn
† Electronic address: l.gu@iphy.ac.cn
‡ Electronic address: ywxie@zju.edu.cn
§ Electronic address: pengrui@fudan.edu.cn
¶ Electronic address: dlfeng@ustc.edu.cn

References

[1] Tan, S. *et al*. Interface-induced superconductivity and strain-dependent spin density waves in FeSe/$SrTiO_3$ thin films. *Nat. Mater.* **12**, 634 (2013).

[2] Song, Q. *et al*. Evidence of cooperative effect on the enhanced superconducting transition temperature at the FeSe/$SrTiO_3$ interface. *Nat. Commun.* **10**, 758 (2019).
